# Large Scale Double-Path Illumination System with Split Field of View for the All-Optical Study of Inter-and Intra-Hemispheric Functional Connectivity on Mice

**DOI:** 10.3390/mps2010011

**Published:** 2019-01-29

**Authors:** Emilia Conti, Anna Letizia Allegra Mascaro, Francesco Saverio Pavone

**Affiliations:** 1 European Laboratory for Non-Linear Spectroscopy, University of Florence, 50019 Sesto Fiorentino, Italy; conti@lens.unifi.it (E.C.); pavone@lens.unifi.it (F.S.P.); 2 Neuroscience Institute, National Research Council, 56100 Pisa, Italy; 3 National Institute of Optics, National Research Council, 50019 Sesto Fiorentino, Italy; 4 Department of Physics and Astronomy, University of Florence, 50019 Sesto Fiorentino, Italy

**Keywords:** wide-field microscopy, all-optical, calcium imaging, channel rhodopsin, optogenetics, functional connectivity, cortical mapping

## Abstract

Recent improvements in optical tools that can perturb brain activity and simultaneously reveal the elicited alterations in the associated regions offer an exceptional means to understand and map the connectivity of the brain. In this work, we exploit a combination of recently developed optical tools to monitor neural population at the meso-scale level and to mould the cortical patterns of targeted neuronal population. Our goal was to investigate the propagation of neuronal activity over the mouse cortex that is triggered by optogenetic stimulation in the contralateral hemisphere. Towards this aim, we developed a wide-field fluorescence microscope that is characterized by a double illumination path allowing for the optogenetic stimulation of the transfected area in the left hemisphere and the simultaneous recording of cortical activity in the right hemisphere. The microscope was further implemented with a custom shutter in order to split the LED illumination path, resulting in a half-obscured field of view. By avoiding the spectral crosstalk between GCaMP6f and channelrhodopsin 2 (ChR2), this system offered the possibility of simultaneous “pumping and probing” of inter-hemispheric functional connectivity on Thy1-GCaMP6f mice.

## 1. Introduction

Investigation of neuronal circuitry and a deep understanding of information propagation in the brain network represent a fascinating goal for neuroscientists. New generation tools, such as indicators of neuronal activity combined with optogenetics actuators, allow for simultaneously revealing brain dynamics and manipulating neuronal activity [1,2,3,4,5]. More in detail, genetically encoded calcium indicators (GECI), like GCaMP6f [6], offer the possibility of targeting specific cell types, populations, or subcellular compartments, allowing for detecting calcium signaling at different scales: from single spines [7] to the entire cortical surface [8,9,10]. Genetically encoded calcium indicators, which are based on fluorescent proteins, provide many advantages deriving from both optical reporting and genetic encoding; thanks for their sensibility and high signal-to-noise ratio GECIs allow for monitoring calcium transients in living cells and organism [11,12,13,14]. Thanks to these features, genetically encoded optical indicators of neuronal activity enabled progress toward this aim to an extent that was unimagined two decades ago. In addition, the use of microbial rhodopsin proteins, such as channelrhodopsin 2 (ChR2), which acts as unitary activated ion pumps or channels that can be activated by specific wavelengths, enable the manipulation of neuronal activity in a targeted cell population with light. The key advantage of optogenetics over conventional stimulation techniques is its ability to control cell-and circuit-specific behavior with high temporal precision [15,16]. Recently, several studies [2,4,5], by exploiting two-photon microscopy, focused their attention on the development of all-optical strategies to control and record neuronal activity patterns in mice. Though two-photon microscopy allows calcium imaging with cellular resolution, to better understand behavior-related neuronal dynamics other imaging techniques, like wide-field fluorescent microscopy, are better suited to mesoscale investigations.

When compared to other techniques, such as two-photon microscopy [17] or fiber photometry [18], which allow for investigating neural activity with high spatial resolution but within a very restricted area (0.01 mm^2^), the great advantage of wide-field imaging is the capability of revealing cortical activity over a large area (tens to hundreds of mm^2^) and following how the neuronal activity propagates throughout different functional regions of the mouse cortex.

By imaging a large field of view over the entire cortex of mouse, wide-field fluorescence microscopy offers the possibility of combining the revelation of neuronal dynamics and optogenetic modulation, allowing the investigation of a neuronal response evoked by light stimulation.

In the present study, we developed a custom made wide-field fluorescence microscope that allows calcium imaging over a large area (5.2 × 5.2 mm^2^) of Thy1-GCaMP6f mouse cortex and the simultaneous laser stimulation of ChR2 through a second illumination path. Towards this aim, the microscope was implemented with a double illumination path with LED and acousto-optic deflectors (AODs). By using AODs as scanning head, we could generate stimulation patterns with sub-millisecond temporal resolution allowing the precise activation of ChR2 expressing cell populations [19,20]. To avoid the unspecific activation of ChR2 transfected neurons due to the spectral overlap between the excitation peak of both GCaMP6f and ChR2, we applied a shutter that was able to split the LED illumination path, resulting in a half-obscured field of view. This system allows revealing the effective GCaMP6f fluorescent signal that is elicited by ChR2 laser activation, in order to investigate inter-hemispheric functional connectivity and intra-hemispheric propagation of cortical dynamic of mouse brain cortex. 

## 2. Materials and Methods

### 2.1. Mice 

All procedures involving mice were performed in accordance with the rules of the Italian Ministry of Health (Protocol Number 183/2016-PR). The mice were housed in clear plastic cages under a 12 h light/dark cycle and were given ad libitum access to water and food. We used a transgenic mouse line from Jackson Laboratories (Bar Harbor, ME, USA), C57BL/6J-Tg (Thy1GCaMP6f) GP5.17Dkim/J (referred to as GCaMP6f mice). The genetically-encoded fluorescent indicator is expressed in excitatory neurons, as controlled by the Thy1 promoter. For these experiments, we used 3 Thy1-GCaMP6f male mice, with an average age of seven months, 25–28 g weight.

### 2.2. Surgery

We performed a thinned skull preparation on the right hemisphere between bregma and lambda to create an optical window for GCaMP6f imaging. After applying the local anesthetic lidocaine 2% (20 mg/mL), the skin over the skull and periosteum was removed. The skull over most of the right hemisphere was thinned using a dental drill. A cover glass and an aluminum head-post were attached to the skull using transparent dental cement (Super Bond, C&S; Sun Medical Company, Moriyama, Japan). We used a dental drill to create a small craniotomy over primary motor cortex (M1) in the left hemisphere, which was identified by stereotaxic coordinates (+0.5 rostro-caudal, −1.75 medio-lateral). For virus injections, we delivered with a glass capillary (diameter of the tip: 50 µm) 1 μl of AAV9-CaMKII-ChR2-mCherry (2.48 × 10^13^ Genome Copy number/mL) 700–900 μm deep inside the cortex. A volume of 0.5 μL was delivered with a glass capillary (diameter of the tip: 50 µm), with pressure at 3 atm, 4 ms of duration, and 7 Hz of frequency. The skin over the skull was then sutured; the animals were placed in a heated cage (temperature 38°) until they fully recovered.

### 2.3. Optogenetic Stimulation and Simultaneous Recording of GCaMP6f Activity

After 30 days, the Adeno-Associated Virus (AAV) injection mice were anesthetized under Zoletil (50 mg/kg) and xylazine (9 mg/kg) and then placed into the stereotaxic holder. A small (2 mm × 2 mm) craniotomy was performed over the injected area to allow for more effective laser stimulation. After placing the mouse under the wide field fluorescence microscope, we performed repeated laser (473 nm) stimulation (1–2 Hz, pulse duration 3–5 ms, pulse train duration 5 s, laser power at the focal plane 5 mW) on the left M1, which was localized by mCherry fluorescence. Spurious activation of ChR2 from the cyan LED (used for GCaMP6f fluorescence excitation) was avoided by blocking half of the illumination path with a shutter that was positioned after the collimator. All of the experiments were performed under deep anesthesia conditions that were characterized by spontaneous cortical activity oscillation <0.5 Hz. As we revealed spontaneous activity with a higher frequency of oscillation, we supplied a further dose of anesthetic and waited until the frequency of oscillation was restored to infraslow level. Both resting state and laser stimulation measurements consist of 40 s registration of cortical activity on deeply anesthetized Thy1-GCaMP6f mice. We applied three stimulation trains for each registration. For each animal, we concatenated four to six 40 s registration after removal of the frames in which the laser was visible.

### 2.4. Wide-Filed Fluorescence Microscope

The custom-made wide-field fluorescence imaging setup was equipped with two excitation sources for the simultaneous imaging of GCaMP6f fluorescence and light-stimulation of ChR2. For the imaging of GCaMP6f fluorescence, a 505 nm LED (M505L3 Thorlabs, New Jersey, NJ, USA) light passed through a band pass filter (482/18 Semrock, Rochester, New York, NY, USA) was deflected by a dichroic filter (DC FF 495-DI02 Semrock, Rochester, New York, NY, USA) on the objective (2.5× EC Plan Neofluar, NA 0.085, Carl Zeiss Microscopy, Oberkochen, Germany). A three-dimensional (3D) motorized platform (M-229 for *xy* plane, M-126 for *z*-axis movement; Physik Instrumente, Karlsruhe, Germany) allowed for sample displacement. The fluorescence signal was selected by a band pass filter (525/50 Semrock, Rochester, New York, NY, USA) and then collected on the sensor of a high-speed complementary metal-oxide semiconductor (CMOS) camera (Orca Flash 4.0 Hamamatsu Photonics, NJ, USA). To perform optogenetic stimulation of ChR2, a 473 nm continuous wavelength (CW) laser (OBIS 473 nm LX 75mW, Coherent, Santa Clara, CA, USA) was overlaid on the imaging path using a second dichroic beam splitter (FF484-Fdi01-25 × 36, Semrock, Rochester, New York, NY, USA). The system has a random-access scanning head with two orthogonally-mounted acousto-optical deflectors (DTSXY400, AA Opto-Electronic, Orsay France). A 20X objective (LD Plan Neofluar, 20×/0.4 M27, Carl Zeiss Microscopy, Oberkochen, Germany) was used to demagnify the image onto a 100 × 100 pixel^2^ area of the sCMOS camera sensor (OrcaFLASH 4.0, Hamamatsu Photonics, NJ, USA). The demagnification, maximizing the acquisition speed, also allows for performing experiments that need a higher acquisition rate, such as voltage sensitive dyes imaging, on the same system. Images (100 × 100 pixels, pixel size 52 μm) were acquired at 25 Hz. Even though our system is capable of performing fluorescence imaging over a large area also in combination with faster neuronal indicator, such as voltage sensitive dyes, cheaper strategies could be applied for calcium imaging. Indeed, an alternative solution to image high sensitive (but slow) calcium indicator, like GCaMP6, could be to use a less expensive camera. At the same time, for ChR2 stimulation, the use of cheaper deflection system, like galvanometric mirror instead of AOD system, could be as well applied if few stimulation sites are needed.

### 2.5. Calcium Imaging

Cortical activity was collected during resting state and optogenetic stimulation of deeply anaesthetized transgenic mice expressing GCaMP6f indicator in excitatory neurons. The fluorescence signal was normalized by extracting the ΔF/F, according to the following formula:

$$\Delta \left[{\mathrm{Ca}}^{2+}\right]\propto \frac{\Delta F}{F\;}=\frac{\left[F_{t}-F_{0}\right]}{F_{0}}$$

where Δ[Ca^2+^] stands for variation of calcium concentration, *F*_0_ is the average of baseline fluorescence intensity, and *F_t_* is the fluorescence issued at a given time. The fluorescence time-traces resulted from the average of the fluorescence signal of all the pixels inside the regions of interest (ROIs) for all the frame of the recording.

### 2.6. Seed Pixel Correlation Map 

Correlation maps for seed pixels located in right M1, secondary motor cortex (M2), primary sensory cortex (S1), and retrosplenial cortex (RS) were generated with mesoscale brain explorer (MBE) software [21] on the processed ΔF/F image stack mediated over the concatenated trains and over mice. A single pixel, within a specific functional area of the mouse cortex is selected as the seed. Pearson correlation (zero lag) is then used to generate a map showing the extent to which brain activity over time at each pixel correlates with that of the seed [22,23].

### 2.7. Correlation Matrix 

The correlation matrices were realized using data from concatenated stimulation train averaged for three mice and then analyzed by MBE software [21]. We calculated the correlation index (*r*) of cortical activity (ΔF/F) between the selected functional regions (see seed pixel analysis) in the right hemisphere. Correlation matrices are generated from the activity of particular brain regions of interest (0.52 × 0.52 mm) within the functional areas of the cortex, according to stereotaxic coordinates, during the resting state and laser stimulation. Each ROI–ROI pair consists of two sets of brain activity with a single correlation value for each pair. Pearson correlation coefficients were computed for each ROI–ROI pair to generate a connectivity matrix that can be used to infer interareal connectivity relationships [23,24,25,26]. This coefficient is calculated with zero lag, without analysing any time delay. The correlation index depicts how the activity in one area correlates with other areas in the matrix, and accounts for both (i) the synchronicity of fluctuations in fluorescence intensity and (ii) time delays with which the same peaks occur in two different areas.

## 3. Results

**Figure 1:**
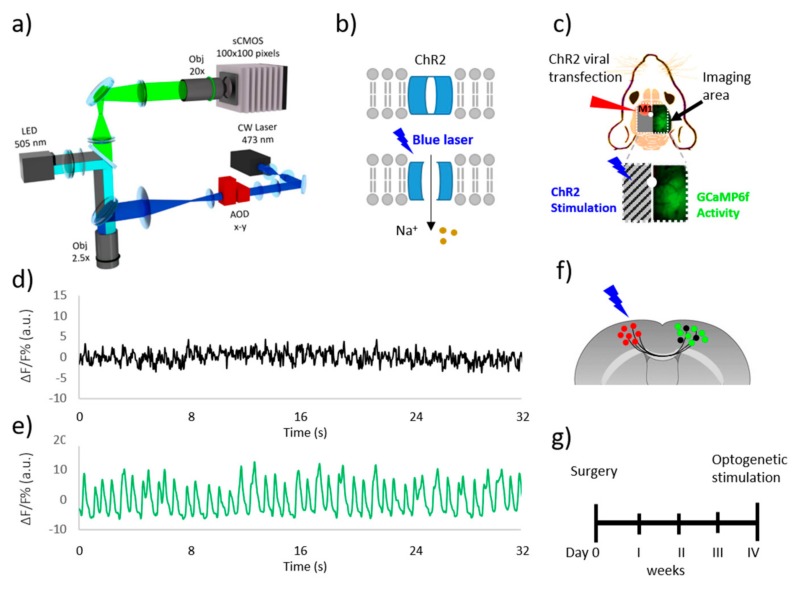
Double-split illumination path. (**a**) Schematic representation of wide-field microscope for the simultaneous laser stimulation and cortical imaging. (**b**) Schematic representation of Channel rhodopsin 2 structure. (**c**) Schematic representation of field of view for inter-hemispheric optogenetic investigation in GCaMP6f mice: ChR2-transfected M1 (red circle) in the left obscured hemisphere is stimulated by the 473 nm blue laser; cortical activity is revealed in the right hemisphere. (**d**) Left hemisphere ΔF/F signal during resting state; (**e**) Right hemisphere ΔF/F signal during resting state; (**f**) Schematic cartoon of interhemispheric connectivity of homotopic regions of the brain cortex via corpus callosum. The red spots represent ChR2-transfected neurons, the green spots represent excitatory neurons expressing the calcium indicator GCaMP6f, the black spots represent inhibitory neurons. (**g**) Experimental timeline. AOD: Acousto-optic deflector.

We took advantage of a custom-made wide field microscope (Figure 1a) for an optogenetic study of intra-hemispheric connectivity on deeply anesthetized Thy1-GCaMP6f mice. A double illumination path characterizes this setup. The primary path used a 505 nm LED to excite the GCaMP6f indicator over a large field of view. In the second path a 473 nm blue laser scanned by AODs is focused on the M1 of the left hemisphere expressing the light-gated cation channel ChR2 (Figure 1b).

Furthermore, we implemented the microscope with a shutter to partially occlude the LED excitation light in order to illuminate the right hemisphere only, keeping the left one in the dark. This technical advancement allowed for the simultaneous recording of cortical dynamics through GCaMP6f fluorescent signal and laser stimulation of ChR2-expressing neurons in the left M1, avoiding the cross-talk excitation of ChR2 with the LED (Figure 1c). By analyzing the recorded signal in both hemispheres (Figure 1d,e), we tested the capability of the shutter to block the LED light. We measured the ΔF/F on two ROIs of the same dimensions (1 × 1 mm^2^) that were placed in the middle of the left and right hemisphere during resting state condition. The signal on the left ROI (obscured side) was very low compared to the ΔF/F measured on the other hemisphere. The signal oscillation revealed in the obscured side, due partially to the light scattering from the adjacent region, resulted in being small (10%) when compared to the signal that was revealed in the other side, proving that the shutter is able to block at least 90% of the excitation. We first tested if the system was able to probe the effective connectivity between the left M1 and the functional areas on the right hemisphere by stimulating the transcallosal pathway. Towards this aim, we injected the M1 of the left hemisphere with AAV9-CaMKII-ChR2-mCherry to induce the expression of ChR2 in excitatory neurons of 3 Thy1-GCaMP6f mice (Figure 1f). After the surgery we waited four weeks to be sure that the transfection was sufficient to evoke a strong cortical activation (Figure 1g).

**Figure 2:**
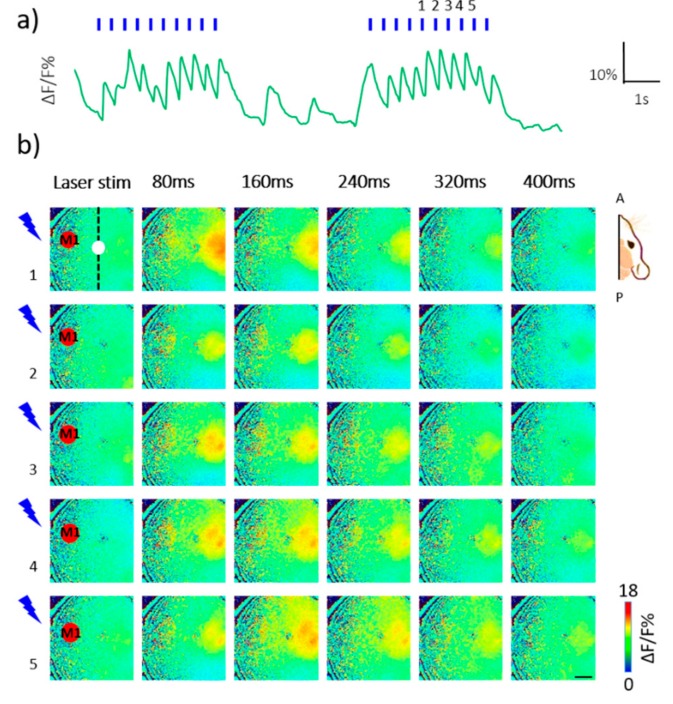
Optogenetic inter-hemispheric stimulation. (**a**) Representative trace of optogenetically elicited cortical response in the right hemisphere during two train of stimulation from a single animal (2 Hz, 5 ms pulse duration); the blue lines represent laser stimulus. (**b**) Representative image sequence from the same recording in A, showing cortical activation in the right hemisphere elicited by 2 Hz pulse of a 473 nm blue laser stimulation in ChR2-expressing M1 in the left hemisphere. Each row corresponds to cortical activity triggered by a single pulse during 2 Hz stimulation. The vertical dashed line indicates the sagittal suture. The white spot on the vertical dashed line indicates the bregma. The red spot in the left hemisphere indicates the transfected M1. A, P: Anterior, Posterior. Scale bar: 1 mm.

For the optogenetic investigation we set the laser power at 14 mW with 5 ms duration pulse at the frequency of 1 or 2 Hz. Laser stimulation of left M1 elicited the neuronal activation in the right hemisphere, evident as a delayed (<80 ms from light stimulus) increment of fluorescent signal. The fluorescence on the right side was reproducibly evoked by laser irradiation (Figure 2). For every pulse, the contralateral side was activated for a few hundredths of milliseconds. The fluorescence levels (measured as ΔF/F) never returned to baseline along the entire stimulus train. The calcium transients reached baseline activity levels when the train was terminated, at the end of each train for all of the stimulations on the three mice. During the inter-stimulus lag, spontaneous activity was always reinstated (Figure 2a). By analyzing the ΔF/F of the cortical registrations, we observed a localized activation of the right hemisphere impinging at first the homotopic region of the cortex (i.e., the M1) and later spreading to other cortical areas that are functionally associated to it. The pattern of activation was reproducible across stimuli, as shown in Figure 2b.

To further explore the spatio-temporal patterns of activation within the right hemisphere elicited by the inter-hemispheric optogenetic stimulation, we analyzed the correlation maps for seed pixels that were located in the right M1, M2, S1, RS during the stimulation.

**Figure 3:**
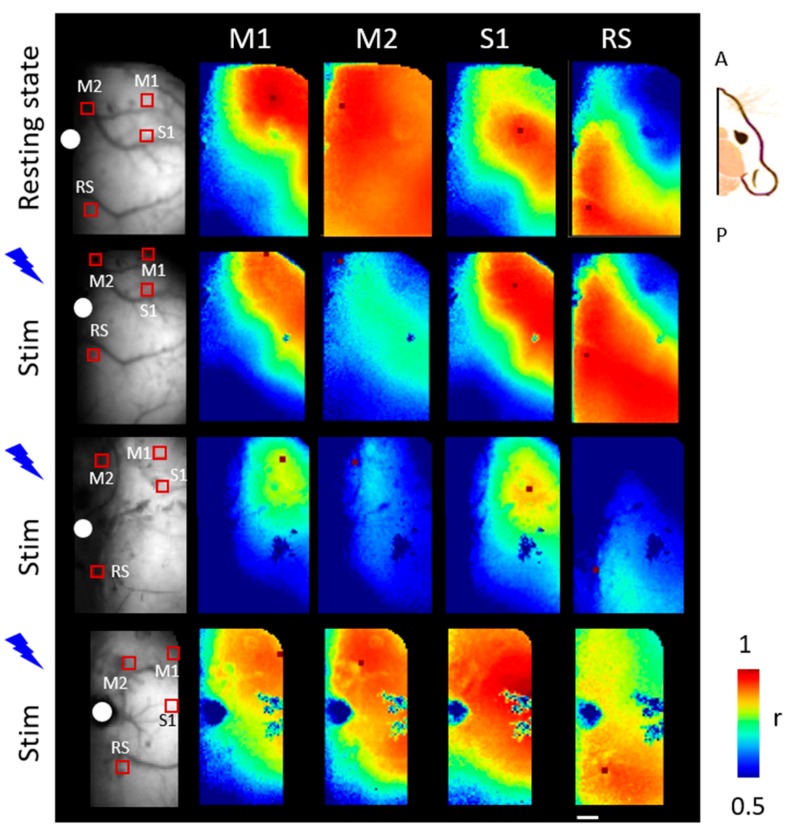
Seed pixel correlation maps of activation during optogenetic stimulation. The panel shows averaged correlation maps for 3 animals, one for each row, during resting state (first row) and optogenetic stimulation. The first and second rows correspond to seed correlation maps of the same mouse during resting state and laser stimulation respectively. In the first column a raw image with the ROIs used for the correlation matrix analysis; the seed is within the ROI. We selected four cortical areas of interest (M1, M2, S1, RS, such as for primary motor cortex, secondary motor cortex, primary sensory cortex and retrosplenial area respectively). The white spot indicates bregma. A, P: Anterior, Posterior. Scale bar: 1 mm.

We positioned a 0.52 mm × 0.52 mm square ROIs on selected functional areas and then we located the seed inside the ROI. We evaluated the correlation between functional areas of the right hemisphere of three deeply anaesthetized Thy1-GCaMP6f mice, both during the resting state and laser stimulation (Figure 3).

We observed in resting state an overall correlated activity regarding the entire hemisphere (see for example Figure 3 first row). By comparing the seed pixel correlation maps that were obtained during optogenetic stimulation, we observed for each seed located in the same ROI cortical patterns of activation specific for the seed location and coherent between animals. More in detail, even though the absolute value of correlation for each seed might change between animals the correlation pattern with the surrounding areas is preserved. Seeds on motor-associated regions (M1 and M2) showed the correlated activity of the sensory areas (S1). Similarly, activation profiles for S1 seed mainly involved motor-associated regions. On the other hand, RS seed maps recruited more-caudal portions of the cortex. This correlation analysis showed that optogenetic stimulation of the contralateral M1 reproducibly triggered the co-activation of functionally related areas in the right cortex.

In order to quantify the correlation between the visualized functional regions of the cortex, we generated correlation matrices. The connectivity values were mediated over the concatenated trains and over mice (see also Table 1). 

**Figure 4:**
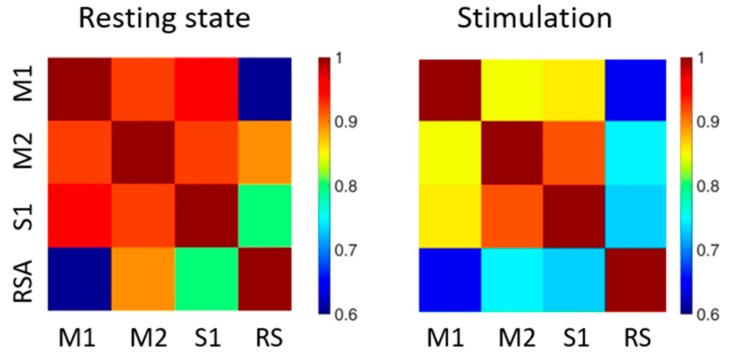
Correlation matrices of functional areas of the cortex. Average correlation matrices during resting state (on the left) and optogenetic stimulation of the contralateral hemisphere (on the right), M1, M2, S1, RS stands for primary motor cortex, secondary motor cortex, primary sensory cortex and retrosplenial area respectively.

We compared two conditions in deeply anaesthetized mice, i.e., resting state (Figure 4, left panel) and during optogenetic stimulation (Figure 4, right panel). In the absence of laser stimulation, the functional areas analyzed showed a high degree of correlation for all regions, and particularly between the motor-sensory areas. During optogenetic stimulation, the overall strength of the connectivity between the analyzed areas was reduced. Indeed, the connectivity values were more differentiated when compared to resting state.

Even if reduced in absolute value, the highest correlations were between the two motor areas (M1 and M2) and between motor and sensory (S1) areas. On the contrary, RS area was the least correlated to the other regions analyzed, specifically during optogenetic stimulation.

## 4. Discussion

The system developed in this study, taking advantage of a double illumination path and of a split illumination, represents a formidable, yet simple, tool to investigate neuronal activity that is elicited by optogenetic stimulation at a mesoscale level, overcoming the limitations due to the overlap of GCaMP6f and ChR2 excitation spectra.

A previous study from Szabo and collaborators [27], avoids the crosstalk between GCaMP indicator and the light gated ChR2, by finely dosing the laser power in a fiber bundle system for a near cellular-resolution optogenetic stimulation coupled to a multimodal imaging device. However, the confined field of view of this approach does not allow mesoscale investigations. On the other hand, previous wide-field studies combining ChR2 actuator with red-shifted voltage-sensitive indicators, even though not affected by spectral overlap, presented some disadvantages, such as the cellular phototoxicity and photobleaching [28,29,30,31]. In addition, voltage-sensitive dyes (VSDs) do not provide cellular specificity and are not applicable for long-term studies. Here, we took advantage of two of the most sensitive actuators and indicators, ChR2 and GCaMP6f, to perform interhemispheric wide-field imaging and stimulation. The spatial separation of the stimulation site and imaging area allowed for recording the cortical activity triggered by optogenetic stimulation over a large field of view, by using a simple wide-field system with small adaptations. In detail, a double illumination path for optogenetics and imaging, and a split field of view, were sufficient in investigating the interhemispheric connectivity between homotopic regions of the cortex. This system could be also suitable for longitudinal studies. This approach is well suited for longitudinal investigation on the effective connectivity between distant areas of the brain, up to the entire cortical surface.

Indeed, by stimulating target region of one hemisphere and simultaneously revealing the triggered neuronal activation on the opposite hemisphere, this custom-made fluorescence microscope allowed for performing interhemispheric studies of functional connectivity. In detail, by dissecting the pattern of activation crossing the corpus callosum it dissected spatial and temporal features of interhemispheric propagation of neuronal signal. In addition, this system allows for visualizing the intra-hemispheric functional connectivity within one hemisphere after optogenetic stimulation of the contralateral side. 

A great advantage of using this poorly invasive approach includes the ability to monitor neuronal activity over a large area in vivo allowing resting state and evoked measurements. Here, we focused on the functional connectivity of targeted functional regions of the cortex (M1, M2, S1, and RS areas). 

By observing the spontaneous activity of awake mice in both hemispheres, a recent study by Vanni et al. [32] showed a functional clustering within the low frequency band (0.1–1 Hz), similar to what we observed with our broad-band seed pixel analysis in anesthetized resting state (see Figure 3, first row).

Here, the stimulation of left ChR2-expressing M1 brings to the focal activation of the homotopic area in the right hemisphere reproducibly between stimulation sessions and among animals, as previously confirmed in other studies [33]. Transcallosal connectivity between the homologous region is the likely driver of this interhemispheric activation. This hypothesis is in agreement with a previous study by Mohajerani and colleagues [34], showing, in resting state, that the corpus callosum is involved in synchronizing bilateral activity between homotopic region.

To our knowledge, the present study shows for the first time the difference in functional correlation patterns within one hemisphere between resting state and following contralateral M1 stimulation. Our preliminary results on the seed pixel correlation analysis suggest that the global functional connectivity is increased in resting state conditions. On the other hand, the contralaterally-evoked connectivity is more clustered and it presents higher connectivity strength between the motor-associated regions (see Figure 4).

Moreover, the cortical propagation that was observed for the selected ROIs resembles the spatial correlation of axonal projections, as observed in previous studies [23,35]. These results confirm that axonal projection structures, as observed in the Allen Mouse Brain Connectivity Atlas (AMBCA, http://connectivity.brain-map.org/ [36]), were related to cortical function. 

## Figures and Tables

**Table 1:** Average Pearson’s correlation coefficients between functional areas (first column) are reported both for Resting State and (second column) and optogenetic Stimulation (third column). Values are presented as mean ± standard error mean (SEM).

Functional Areas	Resting State	Stimulation
M1/M2	0.85 ± 0.06	0.92 ± 0.005
M1/S1	0.85 ± 0.07	0.94 ± 0.01
M1/RS	0.64 ± 0.02	0.62 ± 0.1
M2/S1	0.92 ± 0.03	0.92 ± 0.005
M2/RS	0.75 ± 0.08	0.9 ± 0.03
S1/RS	0.73 ± 0.05	0.81 ± 0.03

## References

[1] Emiliani V., Cohen A.E., Deisseroth K., Hausser M. (2015). All-Optical Interrogation of Neural Circuits. J. Neurosci..

[2] Packer A.M., Russell L.E., Dalgleish H.W., Hausser M. (2015). Simultaneous all-optical manipulation and recording of neural circuit activity with cellular resolution *in vivo*. Nat. Methods.

[3] Ju N., Jiang R., Macknik S.L., Martinez-Conde S., Tang S. (2018). Long-term all-optical interrogation of cortical neurons in awake-behaving nonhuman primates. PLoS Biol..

[4] Zhang Z., Russell L.E., Packer A.M., Gauld O.M., Hausser M. (2018). Closed-loop all-optical interrogation of neural circuits in vivo. Nat. Methods.

[5] Forli A., Vecchia D., Binini N., Succol F., Bovetti S., Moretti C., Nespoli F., Mahn M., Baker C.A., Bolton M.M. (2018). Two-Photon Bidirectional Control and Imaging of Neuronal Excitability with High Spatial Resolution In Vivo. Cell Rep..

[6] Chen T.W., Wardill T.J., Sun Y., Pulver S.R., Renninger S.L., Baohan A., Schreiter E.R., Kerr R.A., Orger M.B., Jayaraman V. (2013). Ultrasensitive fluorescent proteins for imaging neuronal activity. Nature.

[7] Palmer A.E., Tsien R.Y. (2006). Measuring calcium signaling using genetically targetable fluorescent indicators. Nat. Protoc..

[8] Wright P.W., Brier L.M., Bauer A.Q., Baxter G.A., Kraft A.W., Reisman M.D., Bice A.R., Snyder A.Z., Lee J.M., Culver J.P. (2017). Functional connectivity structure of cortical calcium dynamics in anesthetized and awake mice. PLoS ONE.

[9] Balbi M., Vanni M.P., Vega M.J., Silasi G., Sekino Y., Boyd J.D., LeDue J.M., Murphy T.H. (2018). Longitudinal monitoring of mesoscopic cortical activity in a mouse model of microinfarcts reveals dissociations with behavioral and motor function. J. Cereb. Blood Flow Metab..

[10] Xiao D., Vanni M.P., Mitelut C.C., Chan A.W., LeDue J.M., Xie Y., Chen A.C., Swindale N.V., Murphy T.H. (2017). Mapping cortical mesoscopic networks of single spiking cortical or sub-cortical neurons. Elife.

[11] Tian L., Hires S.A., Mao T., Huber D., Chiappe M.E., Chalasani S.H., Petreanu L., Akerboom J., McKinney S.A., Schreiter E.R. (2009). Imaging neural activity in worms, flies and mice with improved GCaMP calcium indicators. Nat. Methods.

[12] Mao T., O’Connor D.H., Scheuss V., Nakai J., Svoboda K. (2008). Characterization and subcellular targeting of GCaMP-type genetically-encoded calcium indicators. PLoS ONE.

[13] Pologruto T.A., Yasuda R., Svoboda K. (2004). Monitoring neural activity and [Ca^2+^] with genetically encoded Ca^2+^ indicators. J. Neurosci..

[14] Reiff D.F., Ihring A., Guerrero G., Isacoff E.Y., Joesch M., Nakai J., Borst A. (2005). In vivo performance of genetically encoded indicators of neural activity in flies. J. Neurosci..

[15] Lorenz-Fonfria V.A., Heberle J. (2014). Channelrhodopsin unchained: Structure and mechanism of a light-gated cation channel. Biochim. Biophys. Acta.

[16] Kim C.K., Adhikari A., Deisseroth K. (2017). Integration of optogenetics with complementary methodologies in systems neuroscience. Nat. Rev. Neurosci..

[17] Tennant K.A., Taylor S.L., White E.R., Brown C.E. (2017). Optogenetic rewiring of thalamocortical circuits to restore function in the stroke injured brain. Nat. Commun..

[18] Li L., Tang Y., Sun L., Rahman K., Huang K., Xu W., Yu J., Dai J., Cao G. (2017). In vivo fiber photometry of neural activity in response to optogenetically manipulated inputs in freely moving mice. J. Innov. Opt. Health Sci..

[19] Crocini C., Ferrantini C., Coppini R., Scardigli M., Yan P., Loew L.M., Smith G., Cerbai E., Poggesi C., Pavone F.S. (2016). Optogenetics design of mechanistically-based stimulation patterns for cardiac defibrillation. Sci. Rep..

[20] Scardigli M., Mullenbroich C., Margoni E., Cannazzaro S., Crocini C., Ferrantini C., Coppini R., Yan P., Loew L.M., Campione M. (2018). Real-time optical manipulation of cardiac conduction in intact hearts. J. Physiol..

[21] Haupt D., Vanni M.P., Bolanos F., Mitelut C., LeDue J.M., Murphy T.H. (2017). Mesoscale brain explorer, a flexible python-based image analysis and visualization tool. Neurophotonics.

[22] Vanni M.P., Murphy T.H. (2014). Mesoscale transcranial spontaneous activity mapping in GCaMP3 transgenic mice reveals extensive reciprocal connections between areas of somatomotor cortex. J. Neurosci..

[23] Mohajerani M.H., Chan A.W., Mohsenvand M., LeDue J., Liu R., McVea D.A., Boyd J.D., Wang Y.T., Reimers M., Murphy T.H. (2013). Spontaneous cortical activity alternates between motifs defined by regional axonal projections. Nat. Neurosci..

[24] White B.R., Bauer A.Q., Snyder A.Z., Schlaggar B.L., Lee J.M., Culver J.P. (2011). Imaging of functional connectivity in the mouse brain. PLoS ONE.

[25] Silasi G., Xiao D., Vanni M.P., Chen A.C., Murphy T.H. (2016). Intact skull chronic windows for mesoscopic wide-field imaging in awake mice. J. Neurosci. Methods.

[26] Chan A.W., Mohajerani M.H., LeDue J.M., Wang Y.T., Murphy T.H. (2015). Mesoscale infraslow spontaneous membrane potential fluctuations recapitulate high-frequency activity cortical motifs. Nat. Commun..

[27] Szabo V., Ventalon C., De Sars V., Bradley J., Emiliani V. (2014). Spatially selective holographic photoactivation and functional fluorescence imaging in freely behaving mice with a fiberscope. Neuron.

[28] Obaid A.L., Loew L.M., Wuskell J.P., Salzberg B.M. (2004). Novel naphthylstyryl-pyridium potentiometric dyes offer advantages for neural network analysis. J. Neurosci. Methods.

[29] Gupta R.K., Salzberg B.M., Grinvald A., Cohen L.B., Kamino K., Lesher S., Boyle M.B., Waggoner A.S., Wang C.H. (1981). Improvements in optical methods for measuring rapid changes in membrane potential. J. Membr. Biol..

[30] Ross W.N., Salzberg B.M., Cohen L.B., Grinvald A., Davila H.V., Waggoner A.S., Wang C.H. (1977). Changes in absorption, fluorescence, dichroism, and Birefringence in stained giant axons: Optical measurement of membrane potential. J. Membr. Biol..

[31] Grinvald A. (1985). Real-time optical mapping of neuronal activity: From single growth cones to the intact mammalian brain. Annu. Rev. Neurosci..

[32] Vanni M.P., Chan A.W., Balbi M., Silasi G., Murphy T.H. (2017). Mesoscale Mapping of Mouse Cortex Reveals Frequency-Dependent Cycling between Distinct Macroscale Functional Modules. J. Neurosci..

[33] Lim D.H., Mohajerani M.H., Ledue J., Boyd J., Chen S.B., Murphy T.H. (2012). In vivo large-scale cortical mapping using channelrhodopsin-2 stimulation in transgenic mice reveals asymmetric and reciprocal relationships between cortical areas. Front. Neural Circuit.

[34] Mohajerani M.H., McVea D.A., Fingas M., Murphy T.H. (2010). Mirrored Bilateral Slow-Wave Cortical Activity within Local Circuits Revealed by Fast Bihemispheric Voltage-Sensitive Dye Imaging in Anesthetized and Awake Mice. J. Neurosci..

[35] Bauer A.Q., Kraft A.W., Baxter G.A., Wright P.W., Reisman M.D., Bice A.R., Park J.J., Bruchas M.R., Snyder A.Z., Lee J.M. (2018). Effective Connectivity Measured Using Optogenetically Evoked Hemodynamic Signals Exhibits Topography Distinct from Resting State Functional Connectivity in the Mouse. Cereb. Cortex.

[36] Oh S.W., Harris J.A., Ng L., Winslow B., Cain N., Mihalas S., Wang Q., Lau C., Kuan L., Henry A.M. (2014). A mesoscale connectome of the mouse brain. Nature.

